# Identification and Structural Elucidation of Galloylated B‐Type Procyanidins From *Rumex obtusifolius* L. Using Enzymatic Digestion, Phloroglucinolysis, and High‐Resolution Mass Spectrometry

**DOI:** 10.1002/jssc.70396

**Published:** 2026-03-29

**Authors:** Silvia Ballert, Marit Gillmeister, Kathrin Kabrodt, Carola Griehl, Wilfried Rozhon, Ingo Schellenberg

**Affiliations:** ^1^ Department of Agriculture, Ecotrophology and Landscape Development Research Group: Analytical and Bioanalytical Sciences Anhalt University of Applied Sciences Bernburg Germany; ^2^ INSTAND e.V. Society for Promoting Quality Assurance in Medical Laboratories Duesseldorf Germany; ^3^ Department of Applied Biosciences and Process Technology Competence Center Algal Biotechnology Anhalt University of Applied Sciences Köthen Germany

**Keywords:** collision‐induced dissociation, electron‐activated dissociation, flavan‐3‐ols, hydrolysis, SCIEX ZenoTOF 7600

## Abstract

Procyanidins from plant extracts demonstrate a broad spectrum of bioactive potential. In order to establish structure‐activity relationships, it is necessary to fully determine the stereochemistry and substitution pattern. Phloroglucinolysis and nuclear magnetic resonance (NMR) are conventionally employed for the analysis of galloylated B‐type procyanidins (PBgals). However, these methods require substantial quantities of the compound at a high degree of purity. In this work, five dimeric galloylated procyanidins isolated from *Rumex obtusifolius* L. roots were analyzed using a two‐pronged strategy. Initially, identification and structure elucidation were performed through the implementation of a novel miniaturized enzymatic digestion with polygalacturonase to effectively remove gallic acid residues. Subsequently, the remaining degalloylated dimeric procyanidins were compared to procyanidin B1, B2, B3, B5, and B7 reference substances based on their retention times. Thus, the *R/S*‐configurations at the C2 and C3 positions of both monomer units and the interflavan linkage were determined as either C4→C8 or C4→C6 in a single analysis. In addition, the position of galloylation was ascertained on the upper or lower unit via high‐resolution MS with collision‐induced dissociation (CID) and electron‐activated dissociation (EAD) fragmentation. This innovative fragmentation technology has been demonstrated to preserve the labile bonds, such as the ester bond to gallic acid. Hence, it yields novel diagnostic fragments for structure elucidation. In silico fragmentation analysis revealed that the radical fragment *m*/*z* 440.0738 only fits galloylation on the upper unit, thereby validating the results of the CID. Furthermore, phloroglucinolysis was equally miniaturized and utilized as an independent method to corroborate the results of enzymatic digestion and LC‐MS/MS. Using these methods, the structural configurations of the five isolated substances were successfully and consistently identified as procyanidin B2‐3‐*O*‐gallate, procyanidin B1‐3‐*O*‐gallate, B2‐3’‐*O*‐gallate, procyanidin B7‐3‐*O*‐gallate, and procyanidin B5‐3’‐*O*‐gallate. To our knowledge, this is the first time that these PBgals are reported in *R. obtusifolius*.

**Abbreviations**
C: (+)‐catechin
C‐ph: (+)‐catechin‐(4α→2)‐phloroglucinol
CID: collision‐induced dissociation
d‐PBgal: degalloylated PBgal
EAD: electron‐activated dissociation
EBC: electron beam current
EC: (−)‐epicatechin
EC‐ph: (−)‐epicatechin‐(4β→2)‐phloroglucinol
ECG: (−)‐epicatechin gallate
ECG‐ph: (−)‐epicatechin gallate‐(4β→2)‐phloroglucinol
Frc: fraction
HR: high‐resolution
HRF: heterocyclic ring fission
IDA: information‐dependent acquisition
KE: kinetic energy
PB: B‐type procyanidin
PBgal: B‐type procyanidin monogallate
PB2‐3’gal: procyanidin B2‐3’‐*O*‐gallate
QM: quinone methide fission
RDA: retro‐Diels–Alder
TOF: time‐of‐flight

## 1 Introduction

Compounds from medicinal or non‐medicinal plants offer boundless prospects for new drug candidates or potential active ingredients for plant protection, due to their chemical diversity [1, 2]. One of the most prominent genera within the Polygonaceae family is *Rumex*, which comprises approximately 200 species distributed globally. They are consumed as vegetables or utilized in traditional medicine and pharmaceutical preparations [3, 4]. The above‐ground parts, leaves, and roots of *Rumex* plants contain various bioactive compounds, including anthraquinones, naphthalenes, flavonoids, stilbenoids, triterpenes, carotenoids, phenolic acids, and vitamins [5, 6, 7]. *Rumex obtusifolius* L., more commonly referred to as broad‐leaved dock, is considered a weed that is widespread in Western and Central Europe, as well as other regions. A variety of phenolic compounds, particularly anthracene derivatives, flavonoids, and procyanidins, have been identified in *R. obtusifolius* plants [7, 8]. Specifically, procyanidins have been found in varying degrees of polymerization and galloylation [9]. Procyanidins exhibit a broad spectrum of bioactivity, with reported antioxidative properties [7, 8, 10], anticancerogenic [11, 12], antibacterial [10], and antifungal activities against human [10] and plant pathogens [10, 13, 14] as well as the ability to inhibit the production of toxins [15]. However, in most studies, biological activity is determined based on crude extracts or extract fractions, for which only the total proanthocyanidin content, mean degree of polymerization, or percentage of galloylation is known [7, 8, 10, 11, 13, 14, 15]. This approach, however, does not take into account the impact of stereochemistry on bioactivity and precise structure‐activity relationships [16, 17].

Dimeric B‐type procyanidins (PBs) are composed of (+)‐catechin (C) and (−)‐epicatechin (EC) monomers that are linked through an interflavan bond from C4 of the upper unit to C8 or C6 of the lower unit. Depending on the monomer combination, for the upper and lower units, procyanidin B1–B4 (4→8) or procyanidin B5–B8 (4→6) are formed, respectively. Galloylation on C3 is common for either unit or both [9, 18]. This results in 16 distinct B‐type procyanidin monogallates (PBgals) and eight digallates. Schmuch et al. [19] determined that procyanidin B gallates isolated from *Rumex acetosa* L. possessed antibacterial properties against the human pathogen *Porphyromonas gingivalis*, causing periodontitis, with procyanidin B2 digallate demonstrating the greatest efficacy, followed by PBs with galloylation of the lower unit. A distinction in antibacterial activity between C4→C8 and C4→C6 linkage was not observed. It was found that the inhibition of the human angiotensin I‐converting enzyme, which is associated with vasoconstriction, was more effective for dimers with a C4→C6 linkage than for those with a C4→C8 linkage. Conversely, for radical scavenging and antiviral properties, the opposite effect concerning the interflavan bond was determined, with an additionally higher activity observed from C4→C8 with galloylation of the lower unit [20, 21]. Chou et al. [12] reported the anticancerogenic properties of procyanidin B2‐3,3’‐digallate and determined, through the investigation of both of its monogallates, that the gallic acid positioned on the lower unit is responsible for the anticancer efficacy. The level of activity of procyanidin B2‐3’‐*O*‐gallate (PB2‐3’gal) matched that of the digallate. These findings underscore the importance of determining both the interflavan bond and gallic acid position in order to accurately derive biological activities.

The most straightforward method of substance identification is the comparison of the retention time of a sample with that of a reference substance, using HPLC. However, galloylated procyanidins are costly and only a few of them are commercially available. Other common approaches for substance identification and structure elucidation include LC‐MS, acid‐catalyzed degradation methods such as thiolysis or phloroglucinolysis, and NMR spectroscopy [9, 22, 23, 24, 25, 26, 27]. Each of these methods has its specific advantages and limitations. LC‐MS is time‐efficient and requires only minute amounts of sample material, but it cannot discern stereochemical positions like the *R/S*‐configurations at C2 and C3 of C and EC. Consequently, the unambiguous identification of the two monomers and their respective dimers is unattainable without reference substances. In addition, common collision‐induced dissociation (CID) distributes energy across the entire molecule, resulting in the cleavage of weak bonds, such as galloylation, first. Consequently, positional determinations of these structures can present a significant challenge [28, 29]. The SCIEX ZenoTOF 7600, equipped with electron‐activated dissociation (EAD), offers a novel fragmentation technology. In the EAD process, an electron beam with adjustable kinetic energy (KE) produces new product ions, where labile bonds may remain intact, allowing the determination of conjugation sites. This technology is predominantly used in drug metabolite identification [29, 30, 31, 32]. Thiolysis and phloroglucinolysis are suitable for crude extracts or fractions to determine the mean degree of polymerization and sum of subunits [26, 33, 34]. However, in order to elucidate the structure of a specific procyanidin, the absence of other procyanidins is imperative. This may require prolonged fractionation and purification protocols to obtain an adequate quantity of a highly pure substance. During phloroglucinolysis, the interflavan bond is cleaved and phloroglucinol binds to the C4 of the upper unit. This approach enables the lower unit to be determined by comparing the retention times to monomeric flavan‐3‐ols. However, reference substances for flavan‐3‐ol phloroglucinol adducts are not commercially available and must be synthesized to clearly identify the upper unit [25, 35, 36]. The interflavan linkage can be ascertained in non‐galloylated procyanidins by cleavage rates. Specifically, cleavage rates of C4→C6 linkage exhibit approximately half the cleavage rate of C4→C8 linkage [26]. Regardless of the linkage type, galloylation additionally reduces cleavage rates, with galloylation of the upper unit exerting a more substantial impact than that of the lower unit, presumably due to steric protection of the interflavan bond. Consequently, similar values are observed for PBgal structures with C4→C8 galloylated upper unit and with C4→C6 galloylated lower unit [37]. Therefore, galloylation complicates the identification of interflavan linkage. Enzymatic digestion of polyphenolic extracts is mostly used in juice and wine production to either increase polyphenol yields or modify polyphenol profiles [38, 39, 40]. The enzyme‐catalyzed digestion of PBgal with, for example, tannase, results in free gallic acid and adduct‐free PB [41, 42, 43]. This enables a comparison with PB reference substances based on their retention time [12]. With this approach, only the determination of the ester bond to gallic acid would remain unknown. NMR requires a multistep process of fractionation and purification to ensure the necessary purity and sample amount for analysis, thereby improving the signal‐to‐noise ratio. In order to prevent rotameric forms from causing broad signals and humps in the spectra, procyanidins should be analyzed at low temperatures. A variety of 1D‐ and 2D‐spectra are employed to comprehensively elucidate the structure [23, 24, 25, 44]. Overall, a high degree of expertise is required to interpret the spectra, especially with additional galloylation. Consequently, a rapid method of investigation that doesn't require high purity or a large sample amount is crucial.

This study aimed to identify and structurally elucidate PBgals isolated from *R. obtusifolius* extract. Substance identification was obtained via high‐resolution (HR) LC‐MS/MS analysis with CID fragmentation. The position of gallic acid was determined using HR LC‐MS/MS with CID and corroborated by EAD fragmentation. This approach enabled the evaluation of the potential challenges and benefits associated with this novel fragmentation technology. Subsequently, the structure elucidation of the monomers and the interflavan bond was executed by an optimized enzymatic digestion of PBgal to PB, followed by a comparison with reference substances. This approach is not limited to the use of pure samples; it is equally applicable to impure or complex samples, such as fractions or crude extracts. In addition, phloroglucinolysis was implemented as an independent method to verify the results determined by enzymatic digestion and LC‐MS/MS. Due to the challenges associated with obtaining sufficient amounts of substances to support identification and potential biological testing, both hydrolyses were significantly scaled down to align with the minimal sample quantities available, broadening the prospective applications of these methods.

## 2 Materials and Methods

### 2.1 Chemicals, Reagents, and Sample

Toluene (UV/IR grade), sodium acetate (99%, p.a.), L(+)‐ascorbic acid (99.5%, p.a.), and acetic acid (100%, p.a.) were purchased from Roth (Karlsruhe, Germany) and phloroglucinol (99%, HPLC grade) from Merck (Darmstadt, Germany). Methanol (HPLC grade), ethyl acetate (HPLC grade), water with 0.1% formic acid (LC‐MS grade), acetonitrile with 0.1% formic acid (LC‐MS grade), methanol (LC‐MS grade), and hydrochloric acid (37%) were obtained from VWR (Bruchsal, Germany). Reference substances for procyanidin B1, B2, B3, C, EC, and (−)‐epicatechin gallate (ECG) were purchased from Extrasynthese (Lyon, France). Reference substances for procyanidin B5 and B7 were kindly provided by the Department of Molecular Food Chemistry and Food Development, Institute of Food and One Health, Leibniz University Hannover [45]. PB2‐3’gal was purchased from BIOZOL (Hamburg, Germany). The enzyme Pectinex Ultra SP‐L was purchased from Special Ingredients Europe (Utrecht, the Netherlands). The sample material was a root extract of *R. obtusifolius* L. (accession number RUM 48; Leibniz Institute of Plant Genetics and Crop Plant Research (IPK) Genebank, Gatersleben, Germany) [46]. The plants were cultivated as described by Gillmeister et al. [14] at Anhalt University (Bernburg‐Strenzfeld, Germany) for a period of nine years prior to harvesting. The cleaned, dried, and cut roots underwent a multistep solid/liquid and liquid/liquid extraction process and final lyophilization, resulting in a dried, crude root extract as described previously [14, 47].

### 2.2 Fractionation and Substance Isolation

The preparative fractionation (Method 1) of the crude root extract was carried out using a preparative HPLC Varian Pro/Prep Star System (Agilent Technologies, Waldbronn, Germany). The separation was accomplished by means of an Agilent ZORBAX Eclipse XDB‐C18 (250 mm × 30 mm; 5 µm) column, which was preceded by a Prep Guard‐C18 (10 mm × 21.2 mm; 10 µm) column. The mobile phase A was composed of water with 0.5% (v/v) acetic acid, while mobile phase B was methanol. The flow rate was 34 mL/min, and detection occurred at 280 nm. The sample was dissolved in eluents of the starting conditions, and injection volumes of up to 9.5 mL were used. The elution profile was as follows: 0–25 min, 10%–47% B (v/v); 25–45 min, 47%–57% B; 45–50 min, 57%–100% B; 50–58 min, 100% B; 58–60 min; 100%–10% B; 60–70 min, 10% B (equilibration). In a total of 107 runs, 88 g of crude extract was separated into 32 fractions (Frc 1–Frc 32), from which the solvent was evaporated using a rotary evaporator. Subsequently, the residues of all fractions were subjected to freeze‐drying. Fractions 5, 6, 7, 10, and 13 contained PBgals and were therefore further examined.

Semi‐preparative normal‐phase separation was used as a second mode of fractionation, exclusively applied to Frc 5 and 10 due to their complexity. These methods were performed using a flash chromatography system, Biotage Selekt (Stockholm, Sweden), Biotage Sfär Silica HC Duo (5 g; 20 µm), and Biotage Sfär Silica Samplet (1 g; 60 µm). Solvent A was toluene, solvent B was ethyl acetate with 5% (v/v) acetic acid, and solvent C was methanol. The flow rate was 9 mL/min and detection occurred from 260 to 330 nm. Up to 25 mg (Frc 5) or 20 mg (Frc 10) were dissolved in 200 µL of starting conditions (see below) and applied onto the Samplet. For Frc 5 (Method 2), the gradient of solvent A to B was as follows: 0–1 min, 22% B (v/v); 1–15 min, 22%–100% B; 15–19.5 min, 100% B; at 19.5 min the solvent was switched to 100% C; 19.5–23.5 min, 100% C. The gradient for Frc 10 (Method 3) started with A to B: 0–1 min, 50% B (v/v); 1–22.6 min, 50%–95% B; 22.6–23.2 min, 95%–100% B; 23.2–26.4 min, 100% B; at 26.4 min the gradient transitioned from B to C; 26.4–33.8 min, 20% C (v/v); 33.8–35.8 min, 100% C. For both fractions, the second collected fraction, designated Flash 2, underwent further fractionation.

Analytical methods and semi‐preparative RP fractionation for either the second (Frc 6, 7, and 13) or third fractionation (Frc 5 Flash 2 and Frc 10 Flash 2) were carried out on an Agilent 1200 Series HPLC system (Waldbronn, Germany). All following methods operated with mobile phase A: water with 0.5% (v/v) acetic acid, mobile phase B: methanol, 30°C column temperature, and detection at 280 nm. The flow rate was 0.8 mL/min (analytical scale) and 3.34 mL/min (semi‐preparative scale), respectively. The analytical survey method (Method 1a) was employed in the analysis of crude extract, fractions of the first fractionation, and final pure substances. An Agilent ZORBAX Eclipse XDB‐C18 column (250 mm × 4.6 mm; 5 µm), protected by a Universal RP (4 mm × 3 mm; Macherey‐Nagel, Düren, Germany) guard column, was utilized in this analysis. The gradient was as follows: 0–6 min, 28%–34% B (v/v); 6–38 min, 34%–100% B; 38–45 min, 100% B; 45–45.1 min, 100%–28% B; 45.1–47 min, 28% B (equilibration). Analytical methods for Frc 5 Flash 2 (Method 2a) and Frc 10 Flash 2 (Method 3a) were performed on a ZORBAX Eclipse XDB‐C8 column (150 mm × 4.6 mm; 5 µm) with a Universal RP (4 mm × 3 mm) guard column. The gradients were as follows: For Method 2a: 0–3 min, 25% B (v/v); 3–10 min, 25%–31% B. For Method 3a: 0–30 min, 20%–35% B; 30–31 min, 20% B (equilibration).

**FIGURE 1:**
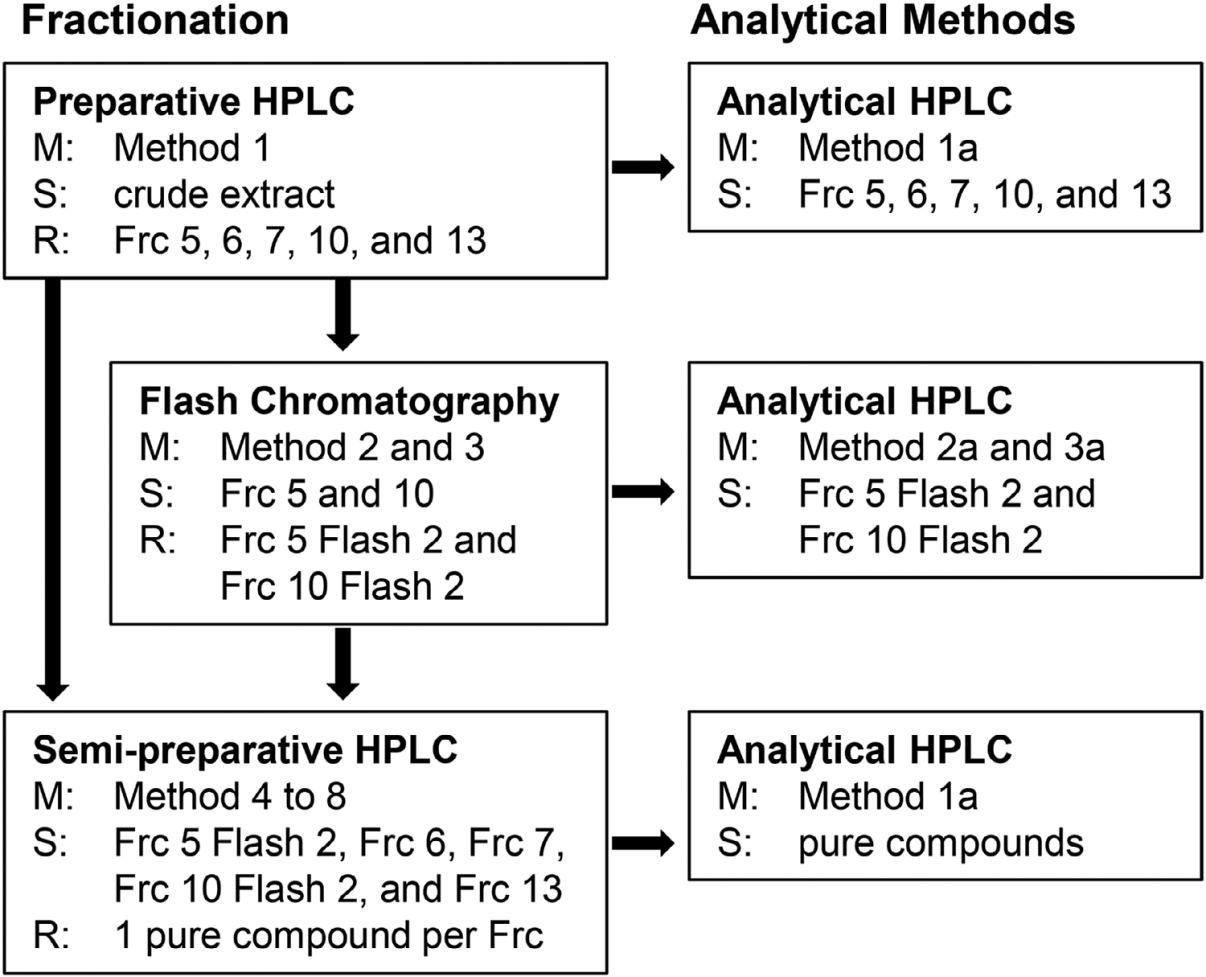
Schematic overview of the fractionation process and subsequent analytical survey of the results. The following information is provided: methods (M), fractionated or analyzed samples (S), and resulting fractions (R).

Semi‐preparative fractionation methods were executed for Frc 5 Flash 2 (Method 4), Frc 6 (Method 5), Frc 7 (Method 6), Frc 10 Flash 2 (Method 7), and Frc 13 (Method 8) using a ZORBAX Eclipse XDB‐C8 (250 mm × 9.4 mm; 5 µm) column and Nucleodur C18 (10 mm × 8 mm; Macherey‐Nagel) guard columns. The gradients for the respective methods were as follows: For Method 4: 0–5 min, 25% B (v/v); 5–10 min, 25%–28% B; for Method 6: 0–17.5 min, 20%–34% B; and for Method 7: 0–18 min, 20%–29% B; 18–19 min, 20% B (equilibration). The methods Method 5 and Method 8 were executed isocratically: 0–15 min, 18% B; and 0–9 min, 38% B, respectively. The fractionation mode was time‐based for Frc 6, 7, and 13, and peak‐based for Frc 5 Flash 2 and 10 Flash 2. This resulted in the collection of a single pure substance per fraction. A schematic overview of the fractionation process is shown in Figure 1.

### 2.3 LC‐MS/MS Analyses

Substance identification and structural annotation were conducted on an LC‐MS/MS system consisting of an Agilent 1260 Infinity II Series coupled with a SCIEX ZenoTOF 7600 QTOF mass spectrometer (Darmstadt, Germany). The separation was carried out on a Phenomenex Kinetex F5 column (150 mm × 2.1 mm; 2.6 µm; Aschaffenburg, Germany), which was equipped with a Phenomenex PFP (4 mm × 2 mm) guard column, with a flow rate of 0.25 mL/min. The mobile phase A was composed of water with 0.1% formic acid, while mobile phase B consisted of acetonitrile with 0.1% formic acid. The gradient for all analyses, aside from phloroglucinolysis products, was as follows: 0–27 min, 10%–68.3% B (v/v); 27–27.1 min, 68.3%–10% B; 27.1–35 min, 10% B (equilibration). As phloroglucinolysis products elute earlier, the gradient was adjusted to: 0–2 min, 10% B; 2–27 min, 10%–45% B; 27–27.1 min, 45%–10% B; 27.1–35 min, 10% B (equilibration). The column was heated to 40°C. For all MS analyses, the gas/source parameters were as follows: ion source gas 1: 40 psi, ion source gas 2: 40 psi, curtain gas: 35 psi, CAD gas: 7, all nitrogen, and the source temperature was set to 440°C. Substance identification was executed in negative ion mode using time‐of‐flight (TOF)‐MS survey full‐scan and information‐dependent acquisition (IDA) MS/MS. The declustering potential was optimized to −140 V, and the ion spray voltage was set to −4500 V. The full scan and IDA covered a mass range from 100 to 1360 Da, with an accumulation time of 0.1 s and 0.025 s, respectively. In addition, for the IDA, a maximum of 15 candidate ions were fragmented in CID mode, with a collision energy of −35 V and a collision energy spread of 15 V for the IDA. The molecular formula was determined via SCIEX OS Formula Finder, based on the TOF‐MS and TOF‐MS/MS spectra. Furthermore, a negative high‐resolution MRM‐mode (MRM HR) was executed with a mass range from 75 to 740 Da. The precursor ion was 729.1461 Da. The collision energy remained at −35 V, with a spread of 15, and accumulation time was set to 0.035 s. In order to potentially obtain additional structural information, electron‐activated dissociation (EAD) and CID were performed in the positive MRM HR. The precursor ion was 731.1592 Da, with a mass range from 75 to 740 Da. The accumulation time was set to 0.035 s, and the ion spray voltage was configured to 5500 V. The optimized declustering potential was determined to be 125 V. For the CID, a collision energy of 35 V and a collision energy spread of 15 V were applied. The specific EAD parameters were as follows: electron beam current (EBC) set to 8000 nA, electron KE set to 8 eV, and reaction time of 30 ms.

### 2.4 Enzymatic Digestion

Pectinex Ultra SP‐L was diluted 1:10 (v/v) with sodium acetate buffer (0.1 mol/L, pH 4.0). In 1.5 mL reaction tubes, 1 µL of sample or reference substance (with concentrations of 5 or 1 mg/mL, respectively, in methanol) was combined with 9 µL of Pectinex dilution and mixed. The tubes were then subjected to an incubation period of 5 h at a temperature of 35°C and a shaking frequency of 400 rpm (Thermomix; Eppendorf, Wesseling‐Berzdorf, Germany). To inactivate the enzyme, 10 µL of methanol were added. For LC‐MS/MS analysis, 10 µL of the mixture were diluted with 190 µL of acetonitrile and 800 µL of water (LC‐MS grade). The sample solution was then filtered through a 0.20 µm Chromafil syringe filter (Macherey‐Nagel). Finally, 2.5 µL of the filtrate were injected for LC‐MS/MS analysis.

### 2.5 Phloroglucinolysis

The method by Kennedy and Jones [34] was modified to significantly reduce the sample amount used for the analysis. A methanolic solution containing 0.1 M HCl, 2.5 g/L phloroglucinol, and 10 g/L ascorbic acid was prepared, along with a 0.2 M sodium acetate solution. For the phloroglucinolysis experiment, 9 µL of the first solution were added to 1 µL of the sample or reference substance (with concentrations of 5 mg/mL or 1 mg/mL, respectively, in methanol). The tubes were then subjected to incubation under shaking at a frequency of 400 rpm for a duration of 10 min. For mild degradation, 30°C was applied, while 50°C was used for full cleavage [25]. The reaction was terminated by adding 10 µL of sodium acetate solution. Then, the degraded sample was mixed with 190 µL of acetonitrile and 790 µL of water (LC‐MS grade). After filtration through a 0.20 µm Chromafil syringe filter, 2.5 µL of the filtrate were injected for LC‐MS/MS analysis.

## 3 Results and Discussion

### 3.1 Substance Isolation

**FIGURE 2:**
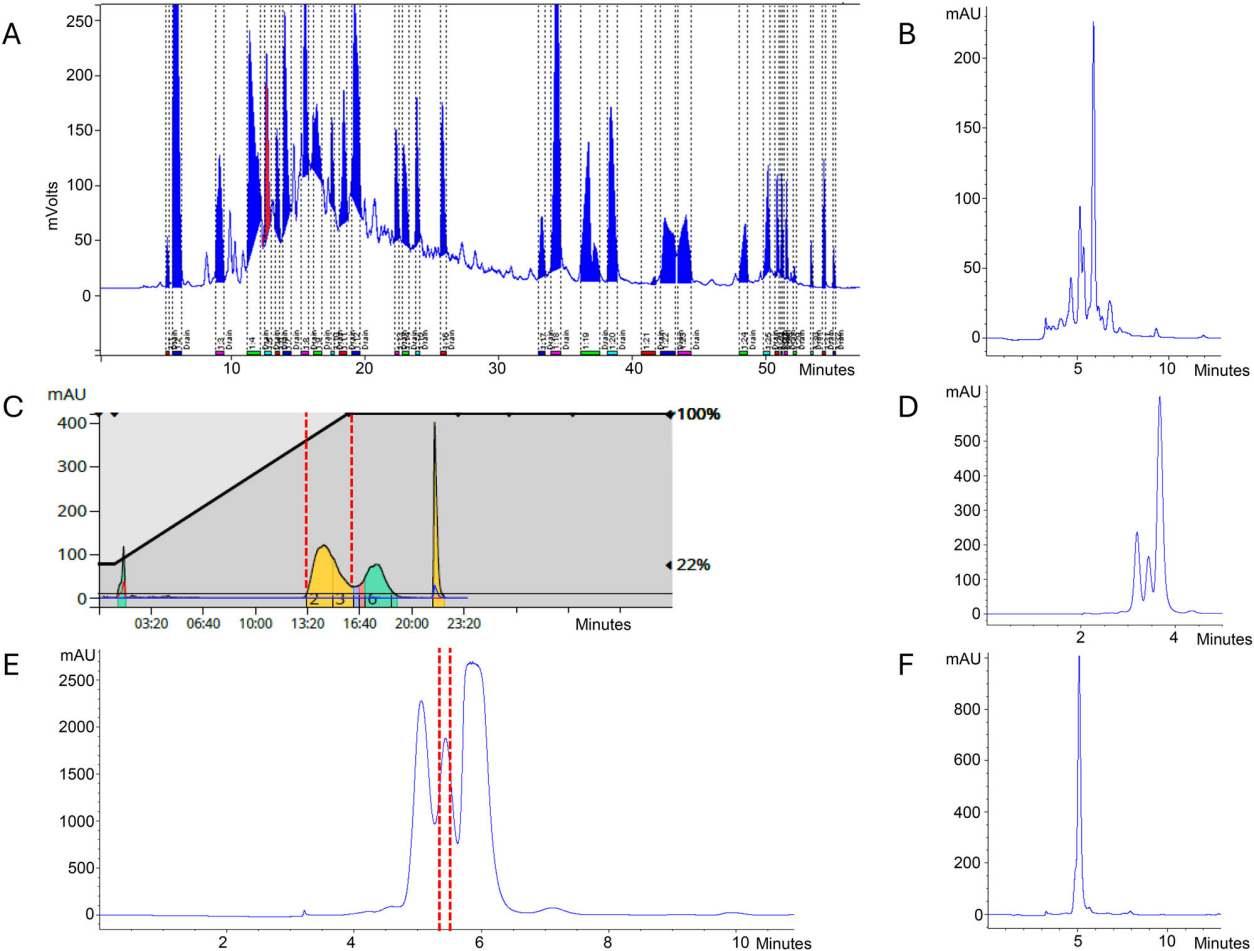
Fractionation process of Fraction 5 (Frc 5): (A) Preparative‐scale fractionation of a crude *R. obtusifolius* extract (Method 1). Frc 5 is marked in red. (B) Frc 5 analyzed using an analytical C18 survey method (Method 1a). (C) Fractionation of Frc 5 via normal‐phase flash chromatography (Method 2). Frc 5 Flash 2, which was investigated further, is marked in red. (D) Analytical C8 survey run of Frc 5 Flash 2 (Method 2a). (E) Fractionation of Frc 5 Flash 2 on a semi‐preparative C8 column (Method 4). The collected fraction is marked in red. (F) Chromatogram of the purified compound in an analytical C18 survey run (Method 1a).

To obtain pure substances, a fractionation process was employed on crude root extract from *R. obtusifolius*, yielding 32 fractions (see Figure 2A). Depending on the complexity of the resulting fraction in Method 1a (Figure 2B), one or two additional fractionation steps were applied. As depicted in Figure 2, two subsequent fractionations were executed in the case of Frc 5. The application of flash chromatography under normal‐phase conditions provided an orthogonal separation. The second peak (Figure 2C) was collected, and the resulting new Frc 5 Flash 2 contained only three substances, as seen in the analytical separation (Figure 2D). In the third step of the procedure, Frc 5 Flash 2 was fractionated again (Figure 2E), after the analytical method was scaled up. The second peak of that separation resulted in Substance 1 with a purity exceeding 90%, based on peak areas at 280 nm (Figure 2F). The results of the fractionation of Frc 6 (Substance 2), Frc 7 (Substance 3), Frc 10 (Substance 4), and Frc 13 (Substance 5) are presented in the Supporting Information (Figures S1–S4). With the exception of Substance 5, isolated from Frc 13, which had a purity of only 83%, all other substances exhibited purities above 90%.

### 3.2 LC‐MS/MS Analyses

**FIGURE 3:**
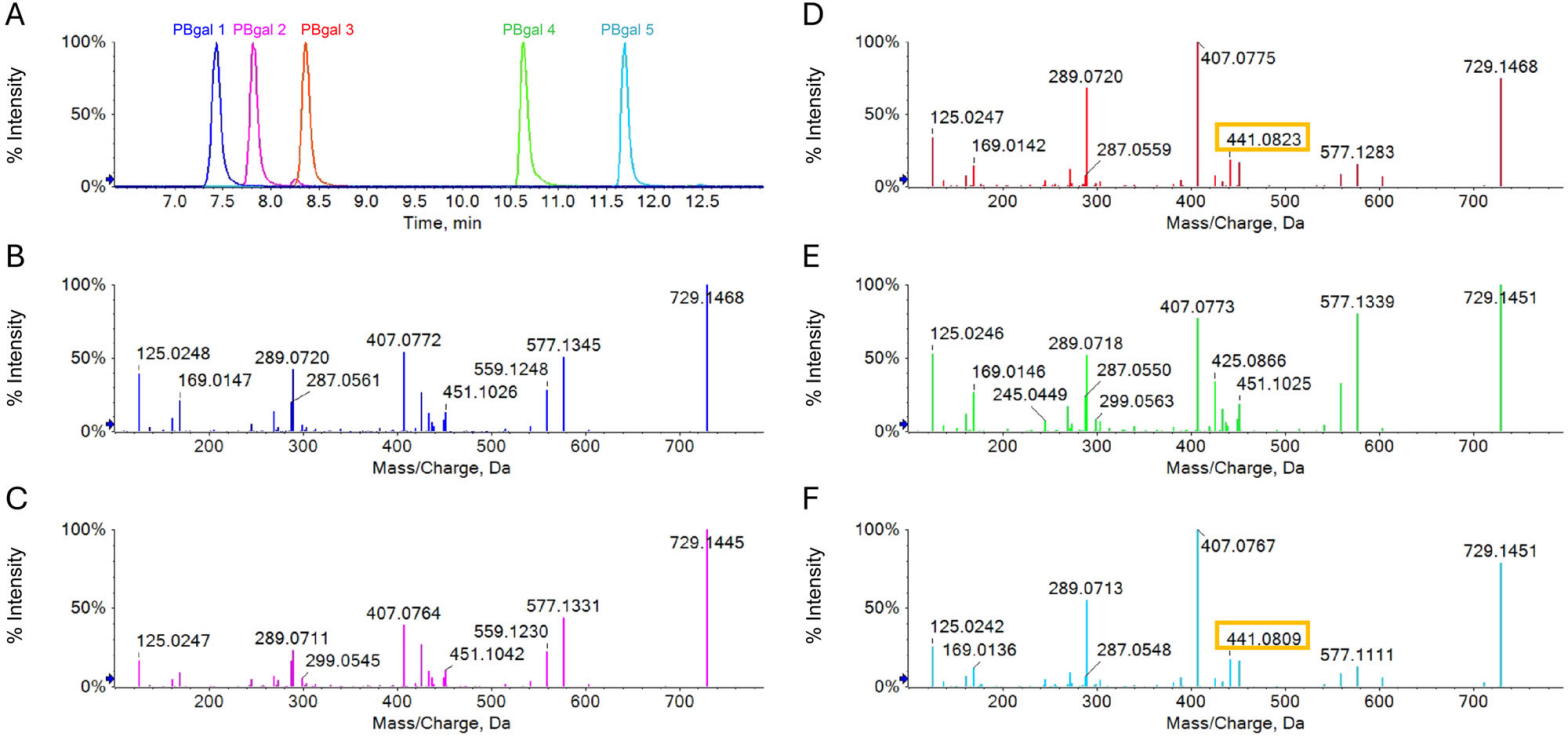
(A) Extracted ion chromatogram (*m*/*z* 729.1461 ± 0.01 Da) of five PBgals and their corresponding MS/MS spectra under negative ionization and collision‐induced dissociation (CID) fragmentation: (B) PBgal 1; (C) PBgal 2; (D) PBgal 3; (E) PBgal 4; and (F) PBgal 5. Fragment 441, specific to quinone methide fission with the gallic acid position on the lower unit, is present in PBgal 3 and PBgal 5 (marked in yellow).

**FIGURE 4:**
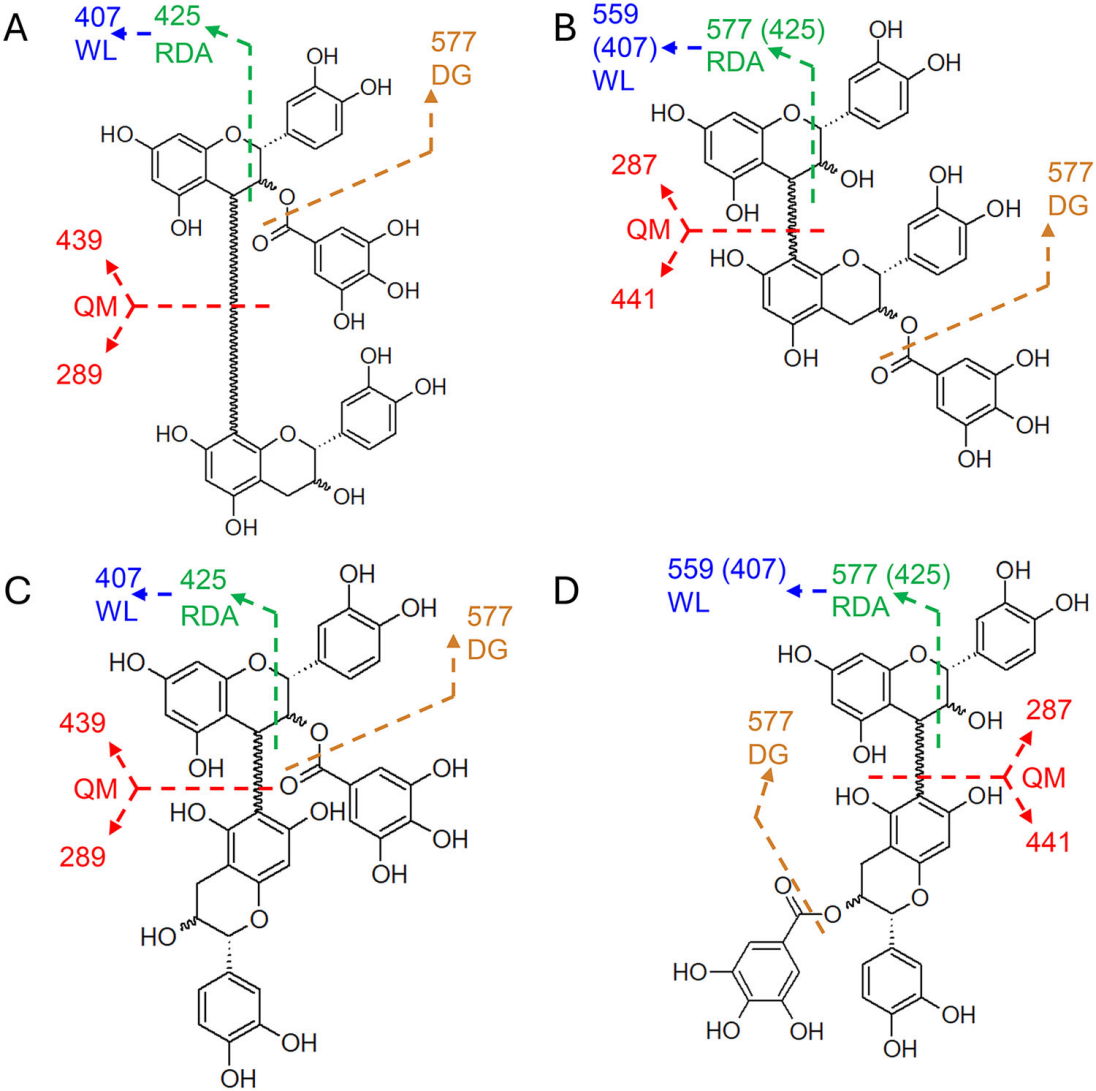
Four stereochemical base structures of PBgal: Monomer linkage C4→C8 with gallic acid bonded to the upper (A) or lower (B) unit, and monomer linkage C4→C6 with gallic acid bonded to the upper (C) or lower (D) unit. Common fragments under negative ionization and CID fragmentation: Degalloylation (DG), quinone methide fission (QM), retro‐Diels–Alder (RDA), and subsequent water loss (WL). Numbers in parentheses assume previous degalloylation.

The initial analysis of all isolated substances was conducted using a full scan with IDA spectra. The molecular formula of all five compounds was determined as C_37_H_30_O_16_ (730.1534 Da) based on TOF‐MS spectra and MS/MS spectra using the SCIEX OS Formula Finder. The extracted ion chromatogram of *m*/*z* 729.1461 (Figure 3A) displayed five distinct peaks. The CID MS/MS spectra for each peak contained the same fragments (Figure 3B–F), which only varied in relative intensity. According to the analysis of the primary fragments, all five substances were identified as PBgal [48]. The presence of nominal masses *m*/*z* 125 and 169 indicates the cleavage of gallic acid or heterocyclic ring fission (HRF) of a monomer. The fragment with *m*/*z* 577 is indicative of the degalloylated PB structure, or retro‐Diels–Alder (RDA) fragmentation prior to degalloylation of the lower unit. The quinone methide fission (QM) is responsible for the cleavage of the interflavan bond, resulting in the formation of either m/z 289 and 439 or m/z 287 and 441, depending on the postion of the galloylated monomer [12, 49]. Furthermore, HRF results in *m*/*z* 451, and RDA fragmentation in *m*/*z* 425 and 407 following water loss [27, 50, 51]. The 16 potential PBgal variations can be grouped into four stereochemical base structures, with each structure presenting four monomer combinations. Mass spectrometry is incapable of distinguishing between the *R/S*‐configurations at C2 and C3 of C and EC [51, 52]. Consequently, it is not possible to differentiate the four compounds per group. The four stereochemical base structures are determined by the interflavan bond, either C4→C8 or C4→C6 linkage, and gallic acid position, derivatized on either the upper or lower unit (Figure 4). Therefore, in order to ascertain the base structure for the five PBgals, it is necessary to determine those fragments that are specific for the interflavan linkage or gallic acid position. Figure 4 illustrates the four base structures in conjunction with the fragmentation pathway for the larger fragments depicted in Figure 3B–F. A comprehensive comparison was conducted of all fragments exhibiting a relative intensity of at least 2% in each spectrum with all four stereochemical base structures via in silico fragmentation of SCIEX OS. The resulting match percentages were found to be identical for all four structures (Table S1). Consequently, the employment of a computer‐based approach proved unsuccessful in determining the interflavan bond or the gallic acid position.

**FIGURE 5:**
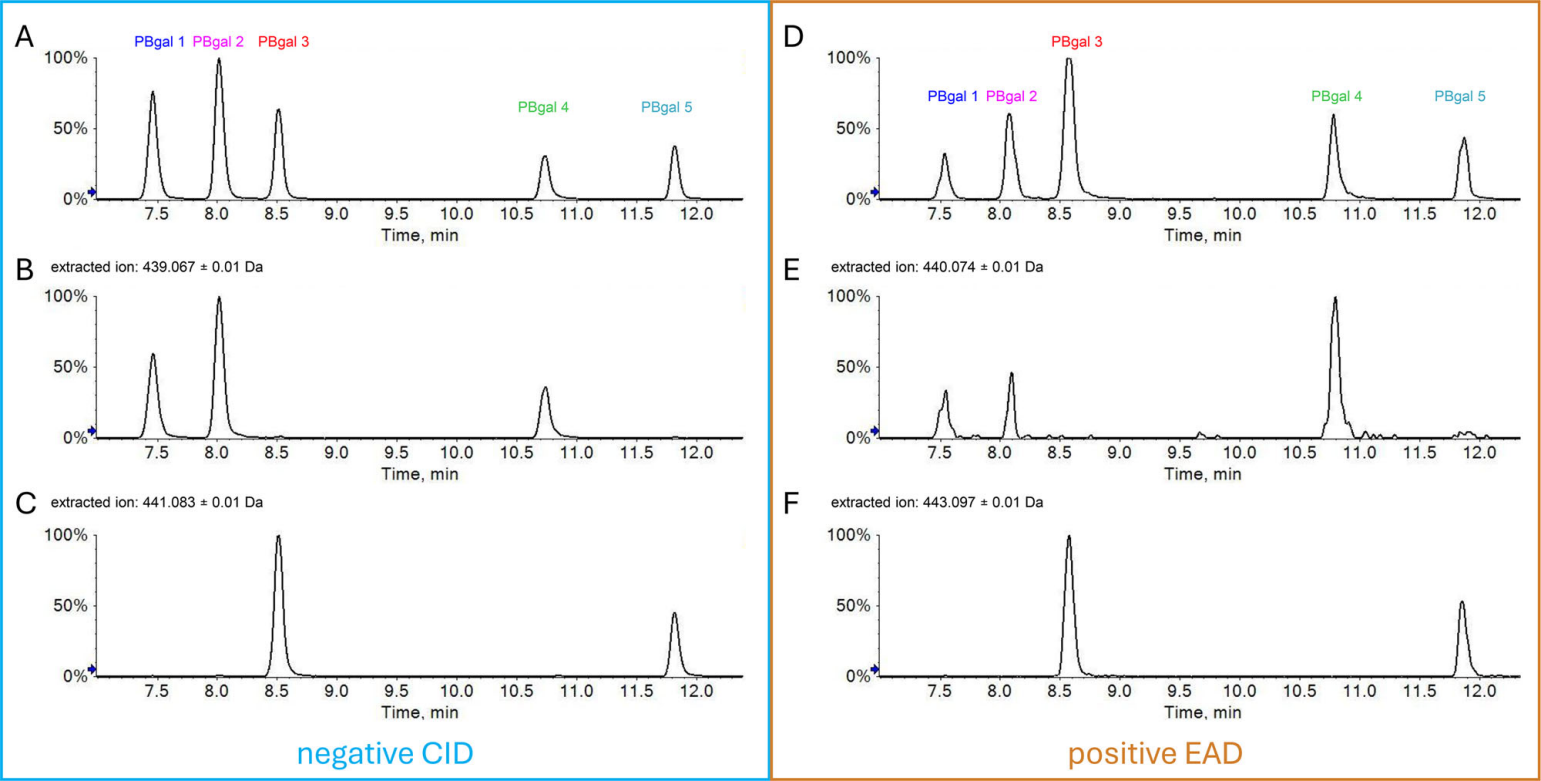
Analysis of all five PBgals in negative CID‐mode (A–C) and positive EAD‐mode (D–F). CID: Extracted ion chromatograms of mother ion *m*/*z* 729.146 ± 0.01 Da (A) and fragment ions *m*/*z* 439.067 ± 0.01 Da (B), indicating galloylation on the upper unit, and *m*/*z* 441.083 ± 0.01 Da (C), indicating galloylation on the lower unit. EAD: Extracted ion chromatograms of mother ion *m*/*z* 731.161 ± 0.01 Da (D) and fragment ions *m*/*z* 440.074 ± 0.01 Da (E), radical ion indicating galloylation on the upper unit, and *m*/*z* 443.097 ± 0.01 Da (F), indicating galloylation on the lower unit.

A thorough examination of the spectra revealed that PBgal 1, isolated from Frc 5 (Figure 3B), PBgal 2 of Frc 6 (Figure 3C), and PBgal 4 of Frc 10 (Figure 3E), exhibited comparable relative intensities, particularly around *m*/*z* 577 and 425. This observation was mirrored by PBgal 3 of Frc 7 (Figure 3D) and PBgal 5 of Frc 13 (Figure 3F) with *m*/*z* 441, suggesting potential structural similarities. The RDA fragmentation to *m*/*z* 425, which is energetically favored for the upper unit [51], could suggest the gallic acid position for PBgals 1, 2, and 4 on the upper unit, as *m*/*z* 425 showed higher intensities than in PBgals 3 and 5. Conversely, the presence of *m*/*z* 441 in PBgals 3 and 5 indicates galloylation on the lower unit [12, 27, 49]. QM of the interflavan bond results in the formation of a fragment for the lower unit that matches the ionized monomer, *m*/*z* 441, in the case of galloylation. The upper unit undergoes a loss of 2H due to the quinone methide formation, resulting in the fragment *m*/*z* 439 in the case of galloylation [53]. This particular process has not been taken into consideration by the automated software approach. The extracted ion chromatograms for the mother ion of PBgal and both fragments are displayed in Figure 5A–C. Despite the low intensity of *m*/*z* 439 in the spectra of Figure 3B–E, the extracted ion chromatogram clearly shows peaks for the identification of galloylation on the upper unit for PBgals 1, 2, and 4. The peaks in the extracted ion chromatogram of *m*/*z* 441 correspond to galloylation of the lower unit for PBgals 3 and 5. However, the interflavan bond could not be determined.

In order to potentially obtain additional characteristic fragments for the structural information of the PBgals, EAD was utilized. During method development, two novel parameters were incorporated: EBC and KE. Typically, LC‐MS/MS analysis of procyanidins is performed in negative mode. Consequently, EAD was also attempted in negative mode. The application of an electron beam to an ion that is already negatively charged can pose a significant challenge and requires a higher level of KE than positive EAD. The method reported by Liu et al. [54] was utilized, with EBC set to 5500 nA and KE set to 24 eV. In addition, EBCs of 8000 nA and KEs of 8, 12, 16, and 20 eV were examined. However, none of the aforementioned settings resulted in the fragmentation of the mother ion, which was evident in the resulting spectra. For single‐charged small molecules, SCIEX suggests the following parameters in positive mode: EBC 7000–8000 nA, KE 8–12 eV, and reaction time 30 ms [55]. For very small and stable molecules, a KE of up to 15 eV may be necessary [30]. The appropriate setting is contingent upon the substance and the objective of the study. Molecules involved in drug metabolism or pharmaceutical chemistry often contain a variety of atom species, functional groups, and conjugation sites. In this context, the use of harder fragmentation with high KE can be advantageous in order to obtain smaller, more intense fragments that are still unique for a specific structure [29, 30, 31, 32]. However, the dimeric structure of PBgal gives rise to fragment repetition, especially within the ring structures consisting exclusively of C, O, and H atoms. Thus, fragments smaller than one monomer may be the result of either unit, rendering them nonspecific. In order to determine the interflavan bond or the conjugation site of gallic acid, it is necessary that the fragment under consideration include the interflavan bond. Therefore, fragments between *m*/*z* 291 and 579, indicative of molecular weights greater than one monomer and less than two monomers, without gallic acid, were of particular interest.

**FIGURE 6:**
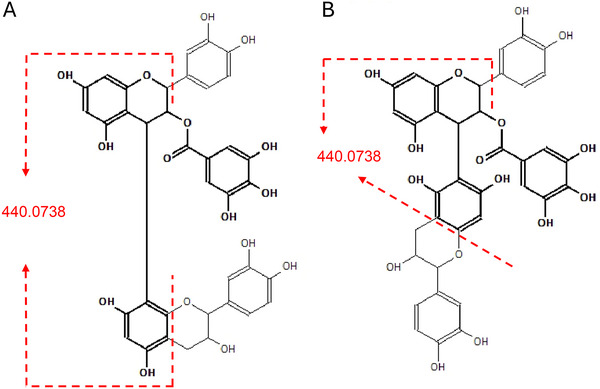
(A) Structure of PBgal with monomer linkage C4→C8 and (B) monomer linkage C4→C6, both with gallic acid bonded to the upper unit. The emphasized structure shows the proposed radical fragment *m*/*z* 440.0738, corresponding to the peak present in PBgal 1, 2, and 4, indicative of galloylation of the upper unit.

In the course of the experiment, settings for EBC of 5500 and 8000 nA, in conjunction with KE levels of 4, 8, 10, and 12 eV, were tested. The EBC of 8000 nA and KE of 8 eV yielded fragments within the desired range. EAD fragmentation can induce the same type of fragmentation as CID, producing similar spectra. Thus, to enhance new EAD‐specific fragments, the positive CID spectrum was subtracted from the EAD spectrum (Figure S5). A comparative analysis of all peaks per spectrum exhibiting a relative intensity of at least 0.2% and in silico fragmentation via SCIEX OS led to radical ion *m*/*z* 440.0738 as a key fragment. This particular fragment was included in the peak list for PBgals 1, 2, and 4, yet it was not present in PBgals 3 and 5. The in silico fragmentation analysis of the given fragment was then executed by matching each of the four base structures of PBgal against the five spectra. This analysis resulted in the exclusive assignment of the fragment to PBgal, with gallic acid esterified on the upper unit. For PBgal galloylated on the lower unit, the peak at *m*/*z* 440 received no fragment assignment. The veracity of both statements is independent of the interflavan bond, whether it is C4→C8 or C4→C6. Figure 6 shows the two interflavan linkage variations of PBgal with galloylation on the upper unit and the proposed fragment formation. Complementary to the extracted ion chromatograms of the negative CID (Figure 5A–C), Figure 5D–F display the extracted ion chromatograms of the PBgal mother ion, radical ion *m*/*z* 440 and 443, for galloylation on the lower unit, of the EAD fragmentation. As previously described by Yao et al., structurally important fragments can exhibit remarkably low peak intensity [29]. The intensity of the EAD signals was at least one order of magnitude lower than that of the CID. Both methods were also executed with the crude extract, where, while still possible, due to the lower PBgal concentration, the intensity difference clearly impacted the EAD signal quality of *m*/*z* 440 (Figure S6). Despite the fact that the EAD analysis ultimately served to validate the CID results, the computer‐based approach that employed in silico fragmentation and the resulting annotation of the gallic acid position on the upper unit based on the EAD fragmentation was successful, in contrast to the CID fragmentation. Unfortunately, EAD did not provide a specific fragment indicative of either interflavan bond. As a result, it remains impossible to fully determine the base structure via LC‐MS/MS.

### 3.3 Enzymatic Digestion

To identify the absolute *R/S*‐configuration at C2 and C3 positions of both monomer units and interflavan linkage between them, without NMR, an enzymatic digestion of PBgal 1–5 was applied. This approach enabled the hydrolysis of the ester bond between gallic acid and PB without the formation of new adducts, as observed in acidic hydrolysis, such as phloroglucinol. Consequently, a comparison with reference substances of PB was possible [12]. As the method described by Agarwal et al. [56] required 1 mg of sample material, the method was scaled down by four orders of magnitude to align with the limited sample material available. Pectinex Ultra SP‐L, a cost‐efficient alternative, was chosen over tannase, which is substrate‐specific for gallic acid. Pectinex contains polygalacturonase and is frequently used in juice production to increase polyphenol yield during extraction, maceration, and pressing processes [38, 39, 57]. The structural similarity between galacturonic acid and gallic acid suggests that polygalacturonase may catalyze the hydrolysis of gallic acid, though with lower activity [58]. In a preliminary test with reference substances, the hydrolysis of EC gallate to EC was proven (Figure S7). Accordingly, all five PBgals were enzymatically digested and then compared to PB reference substances via LC‐MS. Figure S8 shows the extracted ion chromatogram of *m*/*z* 577.1352 for PB, with Figure S8A depicting the peaks for the reference substances, procyanidin B1, B2, B3, B5, and B7. Figure S8B–F are overlaid with the signal of enzymatically digested and degalloylated PBgal 1–5 (d‐PBgal 1–5). Based on the retention time, the procyanidin structure of PBgal 2 was identified as procyanidin B1. PBgal 1 and PBgal 3 were both derived from procyanidin B2, indicating different gallic acid positions. It was determined that all three monomers were linked through C4→C8. The procyanidin structure of PBgal 4 was classified as procyanidin B7, and that of PBgal 5 as procyanidin B5. Both structures exhibited a C4→C6 linkage. These results are consistent with previous reports of procyanidins B1, B2, B3, and B7 in *R. obtusifolius* [9] and procyanidin B5 in *Rumex acetosa* L. [59]. The presence of galloylated forms was therefore anticipated. In addition, procyanidin B6 and B8 are sterically unfavorable and, as a result, more improbable [25]. With a sample of only 5 µg of the substance, all structural information, aside from the gallic acid position, was determined. In conjunction with the findings from the LC‐MS/MS, the five PBgals were identified as follows: procyanidin B2‐3‐*O*‐gallate (1), procyanidin B1‐3‐*O*‐gallate (2), PB2‐3’gal (3), procyanidin B7‐3‐*O*‐gallate (4), and procyanidin B5‐3’‐*O*‐gallate (5). Previous studies have found PB2‐3’gal and procyanidin B5‐3’‐*O*‐gallate in extracts from the aerial parts of *Rumex acetosa* [59], while procyanidin B2‐gallate, without positional specification of gallic acid, has been reported in *Rumex sanguineus* root extract [60]. To date, to our best knowledge, there have been no reports on the five identified procyanidins in *R. obtusifolius* specifically, or in root extracts of *Rumex* spp. in general.

The use of the same organic solvent for both digestion and MS analysis, the reduction of dilution, and the optimization of the MS method with MRM HR instead of full scan IDA have the potential to further reduce the required sample amount. This would accelerate the process and enhance its sensitivity beyond what is achievable with NMR. In addition, the efficacy of this method extends to digallates. Importantly, the use of retention time and MS signal as identifying factors renders the necessity for compound purity nonessential, provided that there are no other procyanidin gallates or digallates present. Consequently, fractions as well as crude extracts could be analyzed by this method.

### 3.4 Phloroglucinolysis

The hydrolysis with phloroglucinol was applied as a reference method to verify the identity of the subunits, their linkages, and gallic acid position. Preliminary tests and method optimizations were executed by digesting reference substances: procyanidin B1, B2, B3, B5, B7, and PB2‐3’gal. To further decrease the amount of sample material needed compared to the procedure described by Esatbeyoglu et al. [25], the sample and reagent volumes were reduced to a minimum. In addition, the concentration of phloroglucinol in the reaction solution was also decreased to match the disproportionately reduced sample amount, as preliminary tests with the original concentration had shown to slow the reaction. The incubation time was increased from 5 min to 10 min in order to approximate hydrolysis rates for mild and full degradation of the C4→C6 and the C4→C8 linkage, as reported by Köhler et al. with mild degradation at 45%–50% and 85%–90%, respectively [37]. The present study found that mild hydrolysis at 30°C resulted in a cleavage rate of 95%–99% for PB1, PB2, and PB3. PB5 and PB7 exhibited cleavage rates of 57% and 68%, respectively, which correspond to the lower hydrolysis rate of the C4→C6 interflavan bond (Table S2). The hydrolysis of PB3 resulted in the formation and retention time determination of (+)‐catechin‐(4α→2)‐phloroglucinol (C‐ph), while the hydrolysis of the other reference substances led to (−)‐epicatechin‐(4β→2)‐phloroglucinol (EC‐ph). Mild phloroglucinolysis of the PBgals yielded the following cleavage rates: PB2‐3’gal 92%, PBgal 1 76%, PBgal 2 48%, PBgal 3 83%, PBgal 4 20%, and PBgal 5 23% (Table S3), clearly indicating a C4→C6 interflavan bond for PBgal 4 and PBgal 5 and a C4→C8 connection for the others. The final structure determination for the five PBgals, based on the full degradation, was therefore as follows: PBgal 1: ECG‐(4β→8)‐EC or PB2‐3‐*O*‐gallate; PBgal 2: ECG‐(4β→8)‐C or PB1‐3‐*O*‐gallate; PBgal 3: EC‐(4β→8)‐ECG or PB2‐3’‐*O*‐gallate (equivalent to the reference substance PB2‐3’gal); PBgal 4: ECG‐(4β→6)‐C or PB7‐3‐*O*‐gallate; and PBgal 5: EC‐(4β→6)‐ECG or PB5‐3’‐*O*‐gallate. The signal of the galloylated upper unit with phloroglucinol of PBgals 1, 2, and 4 occurred at the identical retention time for all three substances. It has been established that all degalloylated procyanidin structures determined by enzymatic digestion possess an EC in the upper unit. Consequently, the signal was identified as ECG‐(4β→2)‐phloroglucinol (ECG‐ph). Galloylation has been reported to reduce the cleavage rate at mild degradation in comparison to the non‐galloylated procyanidin. In this instance, galloylation of the upper unit exerts a more substantial impact due to the steric protection of the interflavan bond [37]. Both statements were corroborated, as all cleavage rates of all PBgals were below the 95% of the non‐galloylated procyanidins. Furthermore, a comparison of PBgal 1 with PBgal 3, and PBgal 4 with PBgal 5, showed a reduced cleavage rate for the first‐mentioned PBgals, respectively, with upper unit galloylation. It appears that the steric hindrance exerts a particularly significant impact on PBgal 2. The results of the phloroglucinolysis serve to validate the results obtained from the LC‐MS/MS and enzymatic digestion analyses. Furthermore, the identity of PBgal 3 was confirmed through a comparison of its retention time with that of the reference substance, PB2‐3’gal.

## 4 Conclusions

This study focuses on the challenges of identification and structure elucidation of natural products, specifically PBgal, in crude plant extracts. Conventional methodologies require the meticulous purification of substances and the collection of substantial sample amounts. Furthermore, the utilization of specialized equipment and expertise is imperative. In order to elucidate the structure of PBgal, it is necessary to determine the *R/S*‐configuration at C2 and C3 of both units, the interflavan bond, and galloylation on C3, either on the upper or lower unit. Rather than employing NMR, this study opted for enzymatic digestion, HR‐MS/MS with CID and EAD fragmentation, and phloroglucinolysis as a reference method. The conjugation site of the gallic acid was determined by LC‐MS/MS. Despite CID's capacity to first cleave labile bonds, such as the esterification of gallic acid, the signals of QM without degalloylation were sufficiently robust to ascertain the position of gallic acid on the upper and lower unit via *m*/*z* 439 and 441, respectively. However, the computer‐based approach did not take QM‐specific fragments into account and, therefore, did not determine the gallic acid position. The EAD fragmentation validated the CID results and provided a characteristic structural fragment of *m*/*z* 440 for galloylation on the upper unit. This radical fragment facilitated the annotation of the conjugation site through a computer‐based approach. Notably, the radical fragment exhibited low signal intensity, a phenomenon that can complicate analysis in complex samples with low substance concentrations, such as crude extracts. The process of enzymatic hydrolysis of the gallic acid ester bond was successfully scaled down. The enzymatic digestion enabled the comparison to PB reference substances, thereby providing the *R/S*‐configuration and interflavan bond within a single analysis. Pectinex, containing polygalacturonase, has been shown to function as a sufficient substitute for tannase. Despite the fractionation and purification steps implemented in this study, experimental findings demonstrated that samples do not necessarily require high purity if subsequent analysis relies on retention time comparison and LC‐MS extraction of the PB ion. Provided that no other PBgals or digallates are present in a given fraction or crude extract, the result will retain its specificity. This method may also be applied to minuscule sample amounts, for example, the collection of a single peak from an analytical LC run. Further development of the method might enable the quantification of PB, both of its PBgal and digallate with a single reference substance via relative response factors. In addition, phloroglucinolysis was meticulously scaled down to accommodate minute sample quantities, thereby enabling the utilization of reference substances to ascertain cleavage rates for mild and full hydrolysis, along with the retention times of phloroglucinol derivatives of the PBgal monomers. As a reference method, it verified the structural determinations of the previously described methods. Consequently, the complete conformation of all PBgal could be determined through a combination of enzymatic digestion and LC‐MS/MS. This information is crucial for obtaining well‐founded results from bioactivity analyses and for drawing conclusions about the structure‐activity relationships of these compounds.

## Author Contributions

**Silvia Ballert**: writing – original draft, writing – review and editing, conceptualization, methodology, investigation, formal analysis, data curation, visualization. **Marit Gillmeister**: writing – original draft, writing – review and editing. **Kathrin Kabrodt**: writing – review and editing, conceptualization, funding acquisition, resources. **Carola Griehl**: writing – review and editing, supervision. **Wilfried Rozhon**: writing – review and editing, funding acquisition, resources. **Ingo Schellenberg**: writing – review and editing, conceptualization, project administration, resources.

## Conflicts of Interest

The authors declare no conflicts of interest.

## Supporting information




**Supporting File**: jssc70396‐sup‐0001‐SuppMat.pdf.

## Data Availability

The authors confirm that the data supporting the findings of this study are available within the article and its supplementary materials.
