# Supplementary material for: Identification and Structural Elucidation of Galloylated B‐Type Procyanidins From Rumex obtusifolius L. Using Enzymatic Digestion, Phloroglucinolysis, and High‐Resolution Mass Spectrometry
Source: J Sep Sci. 2026 Mar 29;49(4):e70396. doi: 10.1002/jssc.70396 (PMC13033918; doi:10.1002/jssc.70396)

Supplementary Material

Identification and structural elucidation of galloylated B-type procyanidins from *Rumex obtusifolius* L. using enzymatic digestion, phloroglucinolysis and high-resolution mass spectrometry

Silvia Ballert[1*], Marit Gillmeister[3], Kathrin Kabrodt[1], Carola Griehl[2], Wilfried Rozhon[1], Ingo Schellenberg[1,3]

[1]Department of Agriculture, Ecotrophology and Landscape Development, Research Group: Institute of Bioanalytical Sciences, Anhalt University of Applied Sciences, Bernburg, Germany

[2]Department of Applied Biosciences and Process Technology, Competence Center Algal Biotechnology, Anhalt University of Applied Sciences, Koethen, Germany

[3]INSTAND e.V., Society for Promoting Quality Assurance in Medical Laboratories, Duesseldorf, Germany

***Correspondence:** Silvia Ballert (silvia.ballert@hs-anhalt.de)

Figure S1: Fractionation process of Fraction 6 (Frc 6) 1
Figure S2: Fractionation process of Fraction 7 (Frc 7) 1
Figure S3: Fractionation process of Fraction 10 (Frc 10) 2
Figure S4: Fractionation process of Fraction 13 (Frc 13) 3
Figure S5: EAD spectra of PBgal 4
Figure S6: Analysis of crude extract in negative CID-mode (A-C) and positive EAD-mode (D-F)... 5
Figure S7: Enzymatic digestion of epicatechin gallate.. 6
Figure S8: Extracted ion chromatograms of m/z 577.1352 ± 0.01 Da 9

Table S1: Comparison of in silico fragmentation of the four base structures of procyanidin B gallates with the five negative CID spectra of procyanidin B gallates... 3
Table S2: Phloroglucinolysis of non-galloylated reference substances 7
Table S3: Phloroglucinolysis of galloylated reference substance and PBgal 8

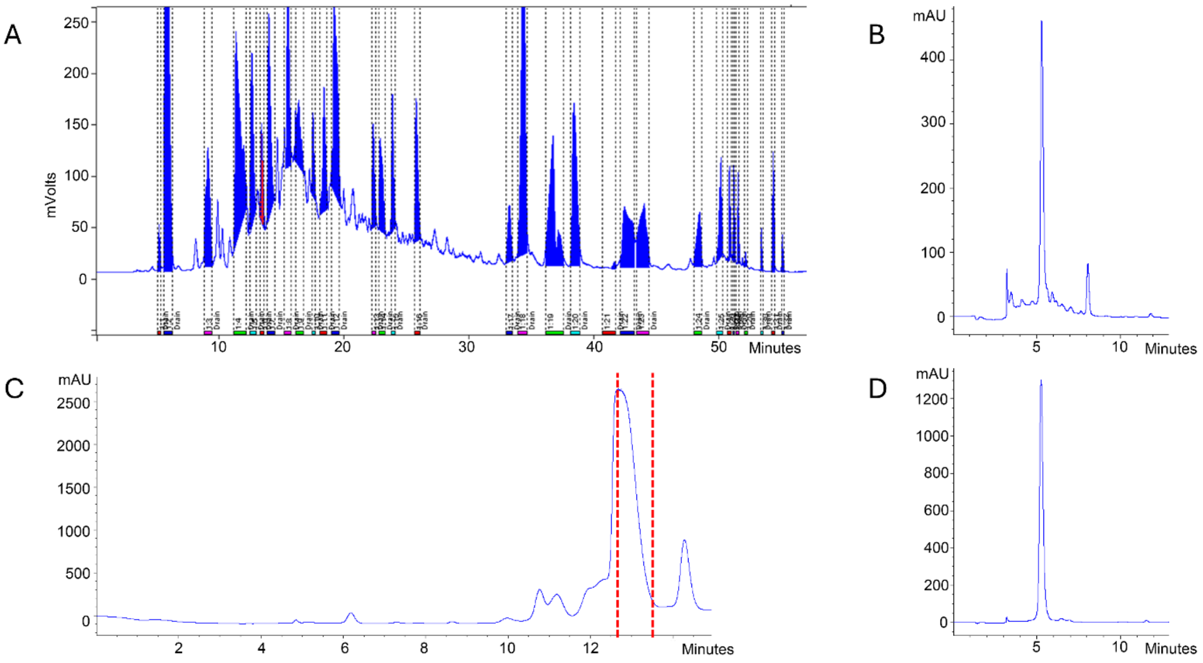

Figure S1: Fractionation process of Fraction 6 (Frc 6): (A) Fractionation of crude extract in preparative scale (Method 1). Frc 6 marked red. (B) Frc 6 run with analytical C18 survey method (Method 1a). (C) Fractionation of Frc 6 on semi-preparative C8 column (Method 5). Collected pure substance marked in red. (D) Pure substance 2 in analytical C18 survey run (Method 1a).

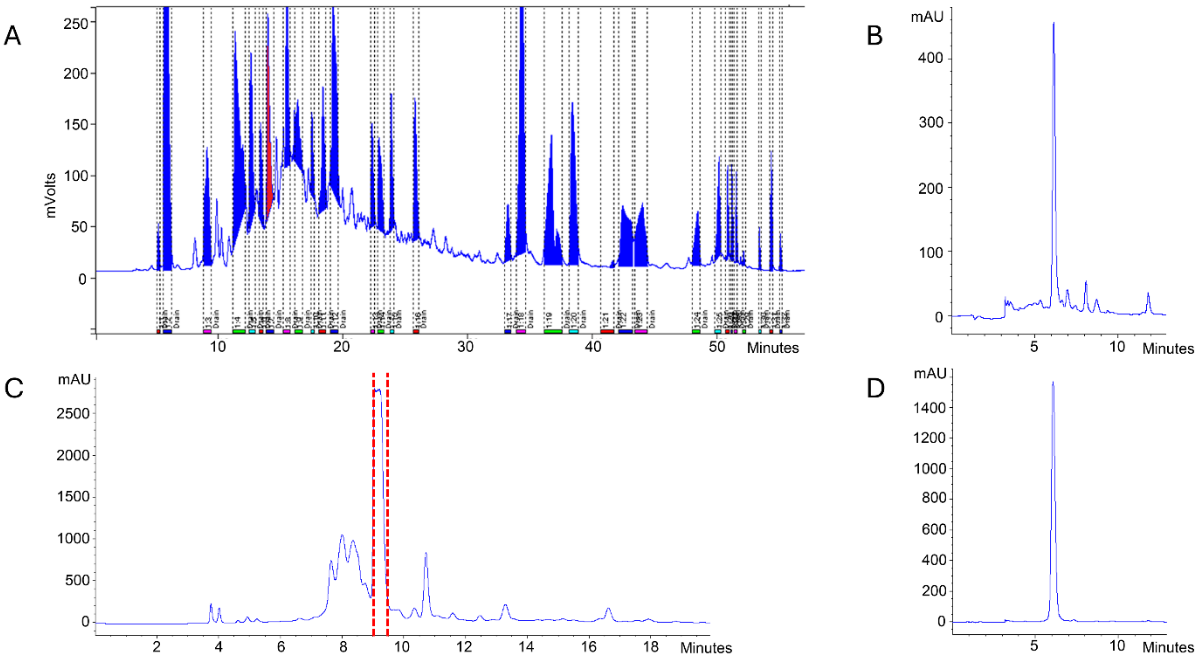

Figure S2: Fractionation process of Fraction 7 (Frc 7): (A) Fractionation of crude extract in preparative scale (Method 1). Frc 7 marked red. (B) Frc 7 run with analytical C18 survey method (Method 1a). (C) Fractionation of Frc 7 on semi-preparative C8

column (Method 6). Collected pure substance marked in red. (D) Pure substance 3 in analytical C18 survey run (Method 1a).

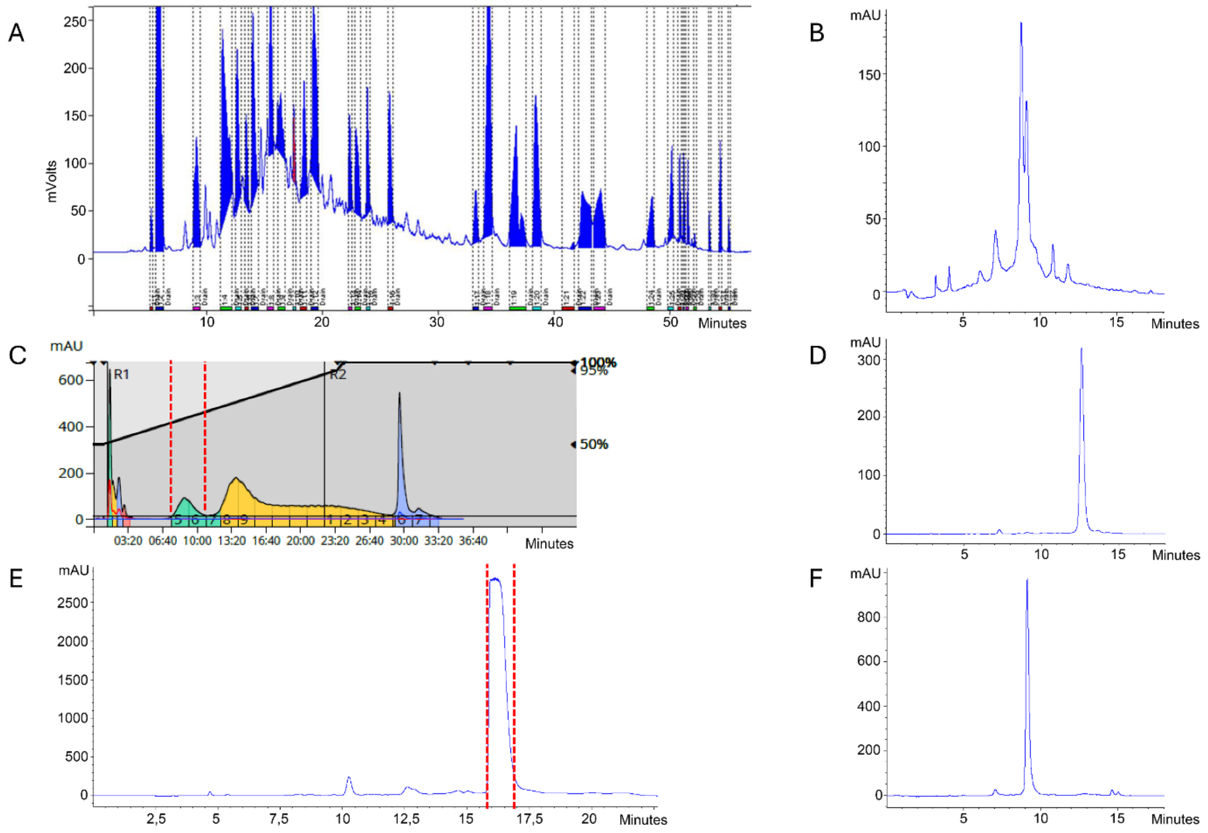

Figure S3: Fractionation process of Fraction 10 (Frc 10): (A) Fractionation of crude extract in preparative scale (Method 1). Frc 10 marked red. (B) Frc 10 run with analytical C18 survey method (Method 1a). (C) Fractionation of Frc 10 via normal phase flash chromatography (Method 3). Further used Frc 10 Flash 2 marked in red. (D) Analytical C8 survey run of Frc 10 Flash 2 (Method 3a). (E) Fractionation of Frc 10 Flash 2 on semi-preparative C8 column (Method 7). Collected pure substance marked in red. (F) Pure substance 4 in analytical C18 survey run (Method 1a).

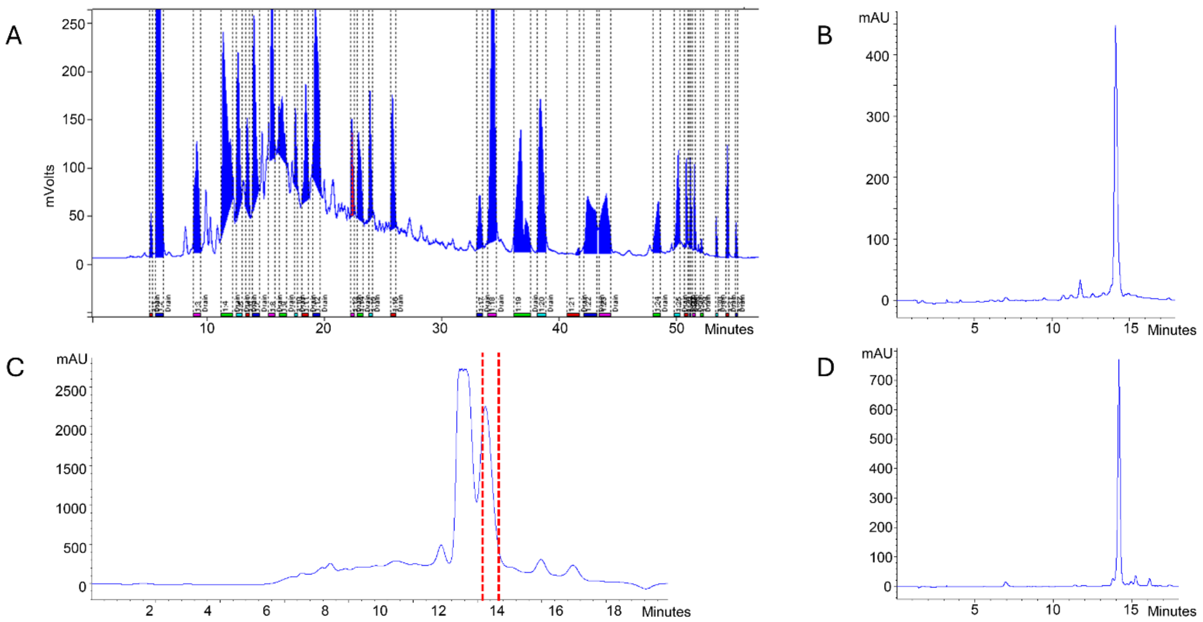

Figure S4: Fractionation process of Fraction 13 (Frc 13): (A) Fractionation of crude extract in preparative scale (Method 1). Frc 13 marked red. (B) Frc 13 run with analytical C18 survey method (Method 1a). (C) Fractionation of Frc 13 on semi-preparative C8 column (Method 8). Collected pure substance marked in red. (D) Pure substance 5 in analytical C18 survey run (Method 1a).

Table S1: Comparison of *in silico* fragmentation of the four base structures of procyanidin B gallates with the five negative CID spectra of procyanidin B gallates. Peak intensity threshold was set to 2%. The match is given in percentage.

structure / compound	C4→C8; galloylated upper unit	C4→C8; galloylated lower unit	C4→C6; galloylated upper unit	C4→C6; galloylated lower unit
PBgal 1	99.6%	99.6%	99.6%	99.6%
PBgal 2	100%	100%	100%	100%
PBgal 3	97.3%	97.3%	97.3%	97.3%
PBgal 4	99.0%	99.0%	99.0%	99.0%
PBgal 5	96.7%	96.7%	96.7%	96.7%

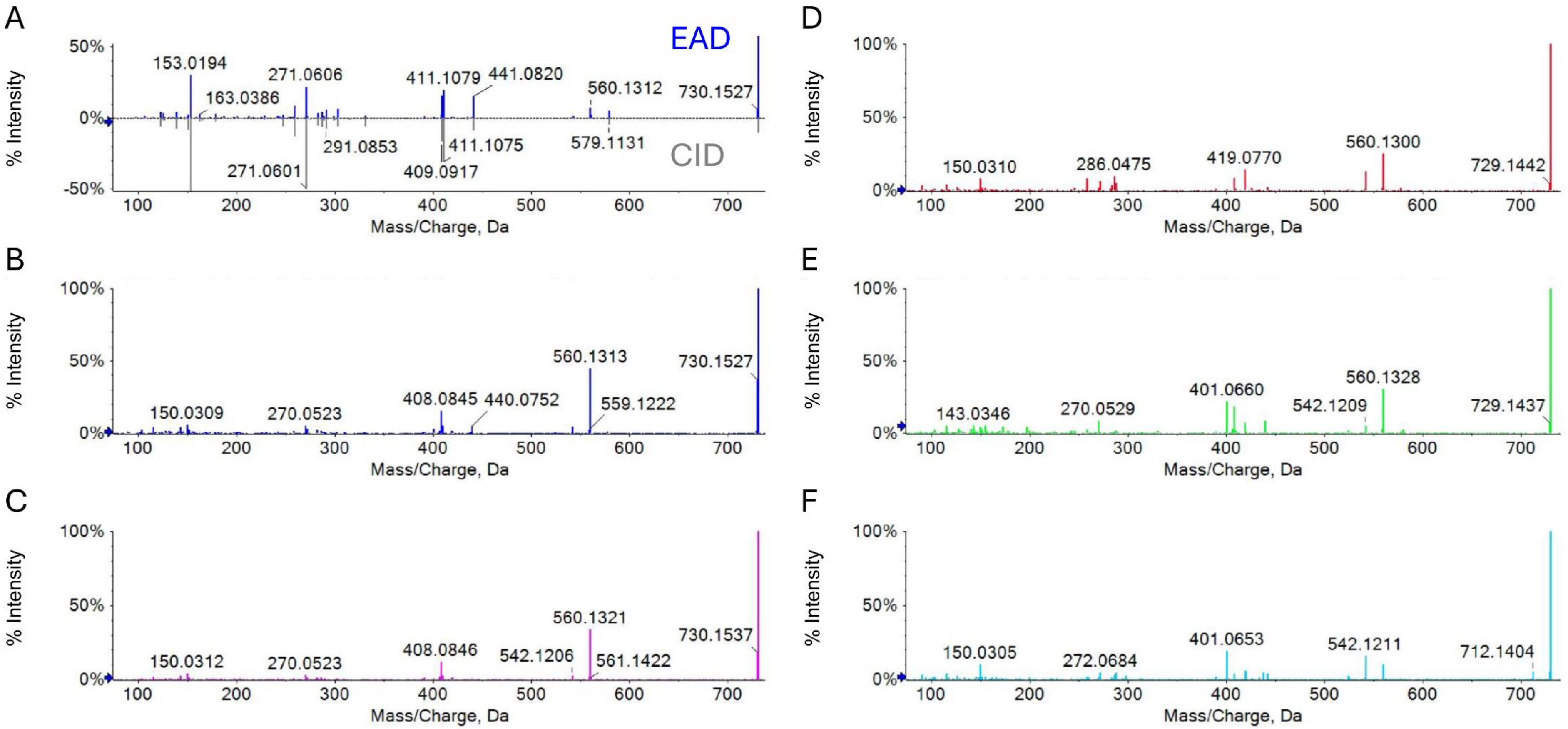

Figure S5: EAD spectra of PBgal: (A) Comparison of EAD (up) and CID (inverted) MS/MS spectra of PBgal 1 under positive ionization. (B-F) EAD MS/MS spectra of procyanidin B gallates (PBgal) cleared of CID fragments via subtraction of CID MS/MS spectra for (B) PBgal 1, (C) PBgal 2, (D) PBgal 3, (E) PBgal 4, and (F) PBgal 5.

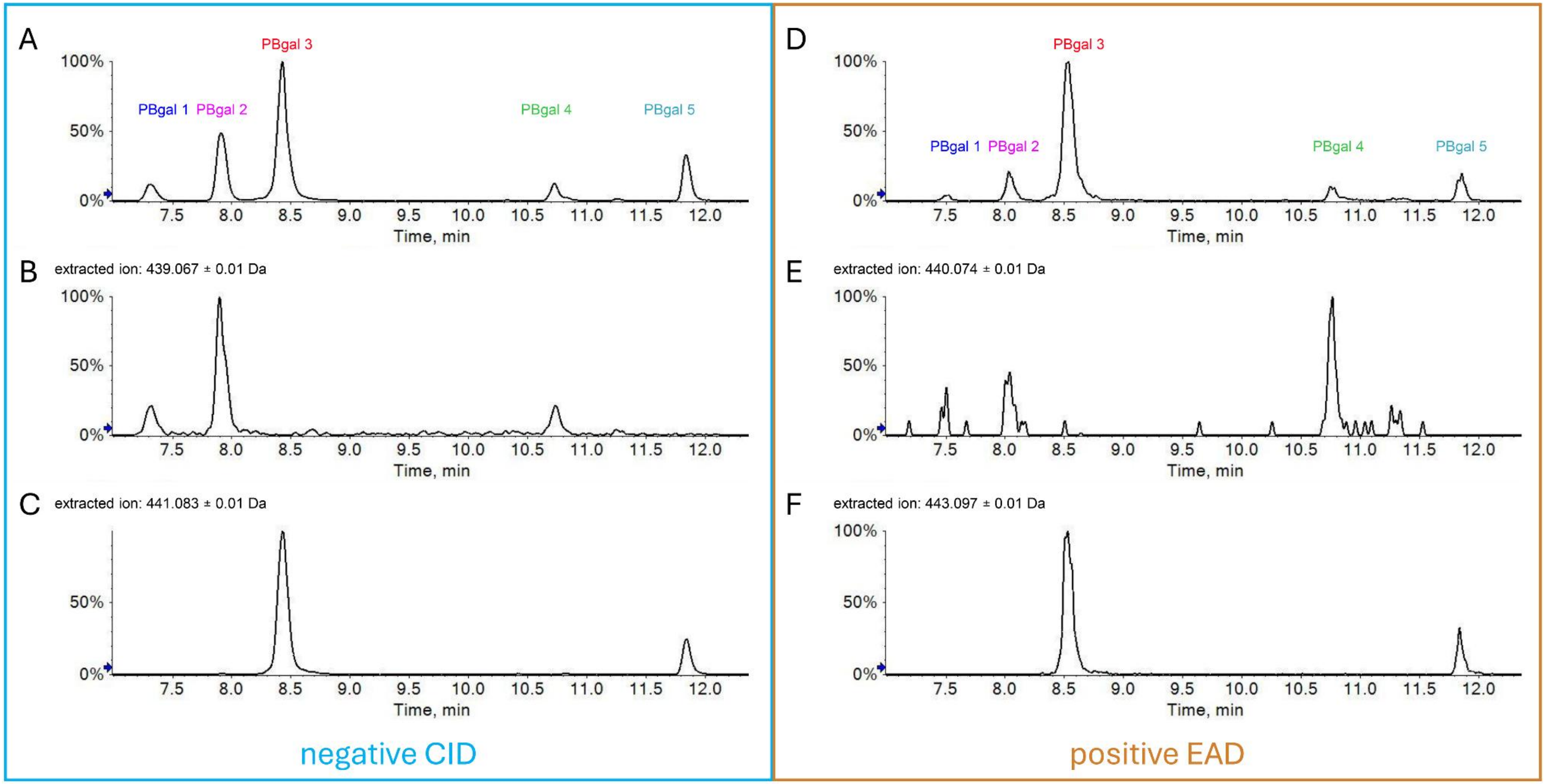

Figure S6: Analysis of crude extract in negative CID-mode (A-C) and positive EAD-mode (D-F). CID: Extracted ion chromatograms of PBgal mother ion m/z 729.146 ± 0.01 Da (A) and fragment ions m/z 439.067 ± 0.01 Da (B), indicating galloylation on the upper unit, and m/z 441.083 ± 0.01 Da (C), indicating galloylation on the lower unit. EAD: Extracted ion chromatograms of PBgal mother ion m/z 731.161 ± 0.01 Da (D) and fragment ions m/z 440.074 ± 0.01 Da (E), radical ion indicating galloylation on the upper unit, and m/z 443.097 ± 0.01 Da (F), indicating galloylation on the lower unit.

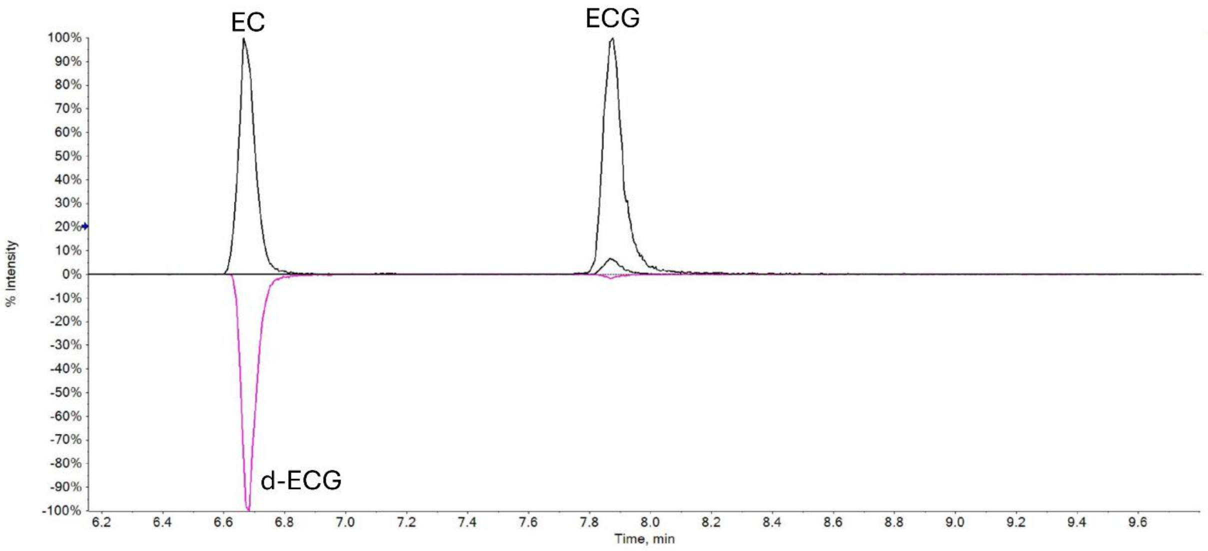

Figure S7: Enzymatic digestion of epicatechin gallate: The overlay of extracted ion chromatograms of reference substances epicatechin (EC; m/z 289.0718 ± 0.01 Da) and epicatechin gallate (ECG; m/z 441.0827 ± 0.01 Da) is shown above in black. The enzymatically digested and degalloylated epicatechin gallate (d-ECG; m/z 289.0718 ± 0.01 Da) is shown inverted in pink. The match of m/z and retention time of d-ECG with EC proves successful hydrolysis from ECG to EC.

Table S2: Phloroglucinolysis of non-galloylated reference substances. Retention time (Rt) and area in counts per seconds (cps) for each substance. Results of their mild (30°C) and full (50°C) phloroglucinolysis with respective cleavage rates and substance identification.

Reference substance		**PB1**		**PB2**		**PB3**		**PB5**		**PB7**	
Retention time [min]		3.27		4.97		4.16		12.86		10.64	
Area before phloro-glucinolysis [cps]		6.21E+5		6.37E+5		7.50E+5		2.31E+5		2.99E+5	
**Phloroglucinolysis**											
Temperature [°C]		30	50	30	50	30	50	30	50	30	50
Peaks after phloroglucinolysis	Rt [min]	Area [cps]	Area [cps]	Area [cps]	Area [cps]	Area [cps]	Area [cps]	Area [cps]	Area [cps]	Area [cps]	Area [cps]
Phloroglucinol	2.02	1.01E+5	8.65E+4	8.23E+4	9.11E+4	9.35E+4	8.70E+4	8.71E+4	8.17E+4	9.11E+4	8.43E+4
C-Phloroglucinol	3.55	-/-	-/-	-/-	-/-	2.27E+5	1.51E+5	-/-	-/-	-/-	-/-
EC-Phloroglucinol	3.41	2.32E+5	1.66E+5	2.93E+5	1.87E+5	-/-	-/-	6.92E+4	7.99E+4	6.43E+4	8.90E+4
C	4.84	2.50E+5	1.89E+5	-/-	5.47E+3	2.85E+5	2.23E+5	-/-	8.25E+2	6.97E+4	1.04E+5
EC	7.67	-/-	-/-	3.05E+5	1.89E+5	-/-	-/-	7.73E+4	8.91E+4	-/-	-/-
Reference substance	s.a.	3.33E+4	8.19E+2	2.33E+4	1.18E+3	8.79E+3	4.85E+2	9.94E+4	1.68E+1	9.69E+4	2.54E+1
Cleavage rate		95%	100%	96%	100%	99%	100%	57%	100%	68%	100%
Identified structure		**EC-(4β → 8)-C**		**EC-(4β → 8)-EC**		**C-(4α → 8)-C**		**EC-(4β → 6)-EC**		**EC-(4β → 6)-C**	

s.a. = see above

Table S3: Phloroglucinolysis of galloylated reference substance and PBgal. Retention time (Rt) and area in counts per seconds (cps) for each substance. Results of their mild (30°C) and full (50°C) phloroglucinolysis with respective cleavage rates and substance identification.

Reference / sample		**PB2-3‘-gal**		**PBgal 1**		**PBgal 2**		**PBgal 3**		**PBgal 4**		**PBgal 5**	
Retention time [min]		10.18		8.3		9.55		10.34		14.1		15.94	
Area before phloro-glucinolysis [cps]		1.84E+5		1.90E+6		3.13E+6		2.69E+6		2.55E+6		1.89E+6	
**Phloroglucinolysis**													
Temperatur [°C]		30	50	30	50	30	50	30	50	30	50	30	50
Peaks after phloroglucinolysis	Rt [min]	Area [cps]	Area [cps]	Area [cps]	Area [cps]	Area [cps]	Area [cps]	Area [cps]	Area [cps]	Area [cps]	Area [cps]	Area [cps]	Area [cps]
Phloroglucinol	2.02	7.09E+4	7.20E+4	8.61E+4	9.20E+4	8.09E+4	8.33E+4	8.43E+4	8.68E+4	8.46E+4	8.69E+4	8.62E+4	8.94E+4
C-Phloroglucinol	3.55	-/-	-/-	-/-	-/-	-/-	-/-	-/-	-/-	-/-	-/-	-/-	-/-
EC-Phloroglucinol	3.41	3.85E+4	5.12E+4	-/-	-/-	-/-	-/-	7.38E+5	6.95E+5	-/-	-/-	2.02E+5	4.39E+5
ECG-Phloroglucinol	8.19	-/-	-/-	8.47E+5	1.15E+6	7.67E+5	1.54E+6	-/-	-/-	2.42E+5	8.67E+5	-/-	-/-
C	4.84	-/-	-/-	-/-	-/-	4.36E+5	7.83E+5	-/-	-/-	1.67E+5	4.68E+5	-/-	-/-
EC	7.67	-/-	-/-	4.70E+5	6.64E+5	-/-	-/-	-/-	-/-	-/-	-/-	-/-	-/-
ECG	13.41	6.06E+4	8.22E+4	-/-	-/-	-/-	-/-	1.44E+6	1.38E+6	-/-	-/-	3.51E+5	8.21E+5
Reference / sample	s.a.	1.50E+4	2.84E+1	4.62E+5	1.28E+4	1.63E+6	3.93E+4	4.67E+5	1.36E+4	2.03E+6	1.24E+5	1.45E+6	2.86E+4
Cleavage rate		92%	100%	76%	99%	48%	99%	83%	99%	20%	95%	23%	98%
Identified structure		**EC-(4β → 8)-ECG; PB2-3'-gallate**		**ECG-(4β → 8)-EC; PB2-3-gallate**		**ECG-(4β → 8)-C; PB1-3-gallate**		**EC-(4β → 8)-ECG; PB2-3'-gallate**		**ECG-(4β → 6)-C; PB7-3-gallate**		**EC-(4β → 6)-ECG; PB5-3'-gallate**	

s.a. = see above

Figure S8: Extracted ion chromatograms of m/z 577.1352 ± 0.01 Da: (A) Procyanidin B (PB) reference substances PB1, PB2, PB3, PB5, and PB7. (B-F) Overlay of the references with the enzymatic degalloylated procyanidin B gallates (d-PBgal) to determine the underlying procyanidin structures of (B) d-PBgal 1, (C) d-PBgal 2, (D) d-PBgal 3, (E) d-PBgal 4, and (F) d-PBgal 5.

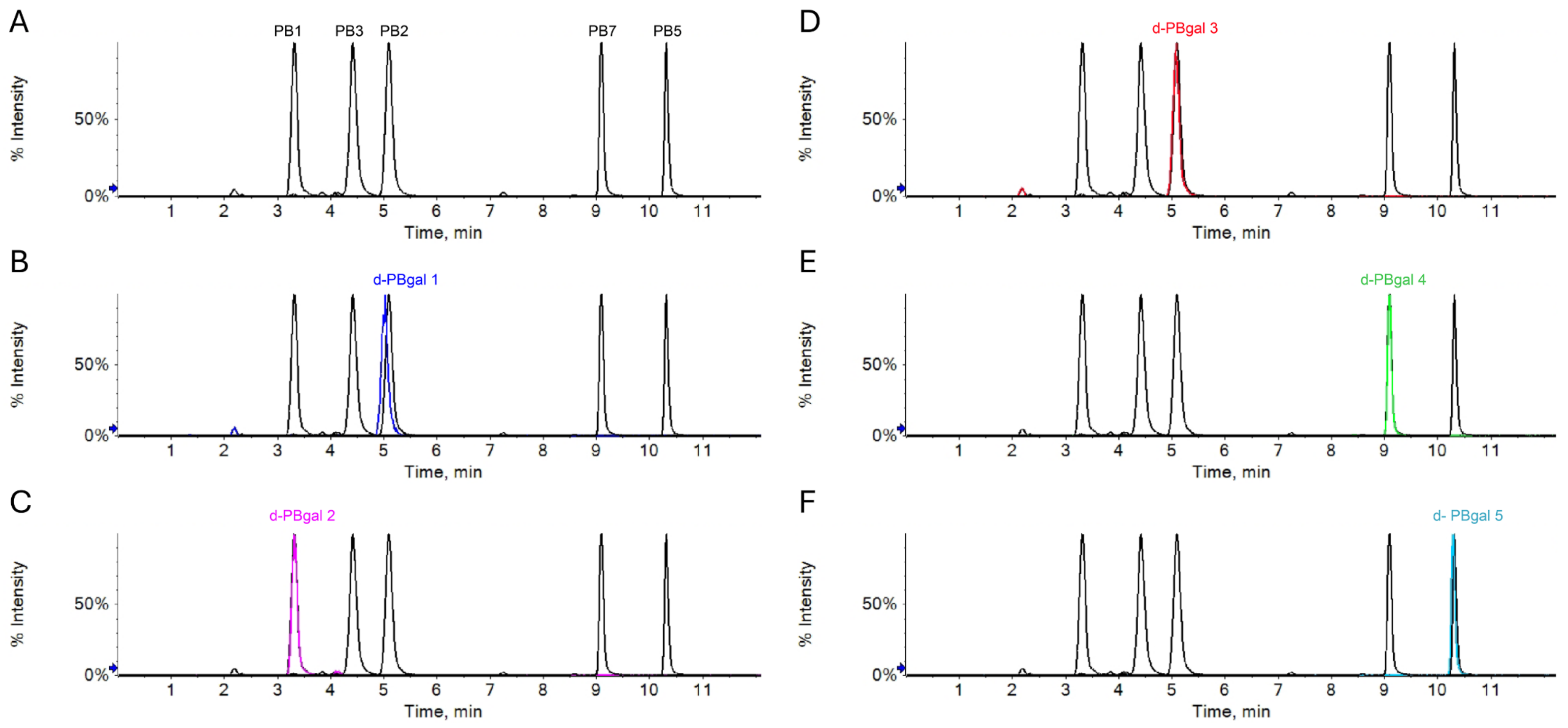

Supplement: Supplementary file 1 — Supporting File: jssc70396‐sup‐0001‐SuppMat.pdf. [file JSSC-49-e70396-s001.pdf]
